# Case report: Analysis of the efficacy and safety of anti-infectious treatment for brain abscess caused by oral anaerobes

**DOI:** 10.3389/fphar.2025.1506879

**Published:** 2025-05-09

**Authors:** Chunfang Tian, Jingxian Liu, Zhiyu Chen, Lixia Li

**Affiliations:** ^1^ Department of Pharmacy, Xin Hua Hospital Affiliated to Shanghai Jiao Tong University School of Medicine, Shanghai, China; ^2^ Department of Pharmacy, Urumqi First People’s Hospital (Urumqi Children’s Hospital), Urumqi, China; ^3^ Department of Clinical Laboratory, Xin Hua Hospital Affiliated to Shanghai Jiao Tong University School of Medicine, Shanghai, China; ^4^ Department of Anesthesiology and SICU, Xin Hua Hospital Affiliated to Shanghai Jiao Tong University School of Medicine, Shanghai, China

**Keywords:** oral anaerobes, brain abscess, metagenomic next-generation sequencing, metronidazole, meropenem

## Abstract

Anaerobic meningitis is relatively rare, and the positivity rate of cerebrospinal fluid (CSF) cultures is exceedingly low, particularly in light of the limited research data regarding bacterial meningitis caused by oral anaerobes. This report presents a case involving a 24-year-old woman who developed fever and headache 32 days after undergoing a cesarean section. The symptoms persisted for 2 weeks, and enhanced nuclear magnetic resonance (NMR) scanning confirmed the suspicion of a brain abscess. Additionally, metagenomic next-generation sequencing (mNGS) of cerebrospinal fluid (CSF) identified several microbial species, including *Porphyromonas gingivalis*, *Prevotella heparinolyticus*, *Fusobacterium nucleatum*, *Parvimonas micra* and *Filifactor alocis*. Bacterial culture of CSF revealed the growth of *Prevotella heparinolyticus*. Following bilateral ventricular external drainage, intracranial lesion resection, and the implantation of an Ommaya reservoir in the right lateral ventricle, cranial decompression treatment was performed. Antimicrobial therapy administered successively over a period of 6 weeks, including vancomycin, meropenem, metronidazole, polymyxin B and ceftazidime, resulting in significant control of the infection. Clinical pharmacists engaged in comprehensive discussions with clinicians regarding the antimicrobial drug regimens and recommended a combined regimen of meropenem and metronidazole. An individualized anti-infective treatment protocol was developed based on therapeutic drug monitoring (TDM), which is anticipated to yield valuable insights for the management of brain abscesses resulting from oral anaerobic bacteria.

## Background

Brain abscesses are focal infections with an estimated incidence of 0.3–0.9 cases per 100,000 inhabitants annually in developed countries, exhibiting a male-to-female ratio of 2:1 to 3:1, with the median age of affected individuals ranging from 30 to 40 years (Sonneville et al., 2017). Jacob Bodilsen et al. (2024) reported an increasing trend in the incidence of brain abscesses attributed to oral cavity bacteria from 2007 to 2020. However, brain abscesses resulting from documented tooth infections remain relatively rare.

Anaerobic bacteria present in the human oral habitat, including *Prevotella* spp., *Fusobacterium* spp., *Bacteroides* spp. and *Peptostreptococcus* spp., are recognized as conditional pathogens. Notably, the majority of meningitis cases caused by these anaerobes are characterized as mixed infections involving multiple anaerobic species (Zhong et al., 2023). The mortality rate among patients with brain abscesses is approximately 25%, and even in cases where patients are cured, 30%–55% experience neurological sequelae, the prognosis for brain abscesses accompanied by ventriculitis is significantly poorer (Takahashi et al., 2020). Due to difficulties in isolating and identifying anaerobic bacteria, relatviely few studies have specifically addressed brain abscesses caused solely by anaerobic pathogens. This article presents a rare case of intracranial infection in a puerperal female patient, where multiple oral anaerobes were identified from the CSF sample through mNGS and culture. It discusses therapeutic measures and options for antimicrobial drug selection in the context of relevant literature, aiming to provide clinicians with valuable insights and support for the treatment of brain abscesses induced by anaerobes.

## Case presentation

**FIGURE 1:**
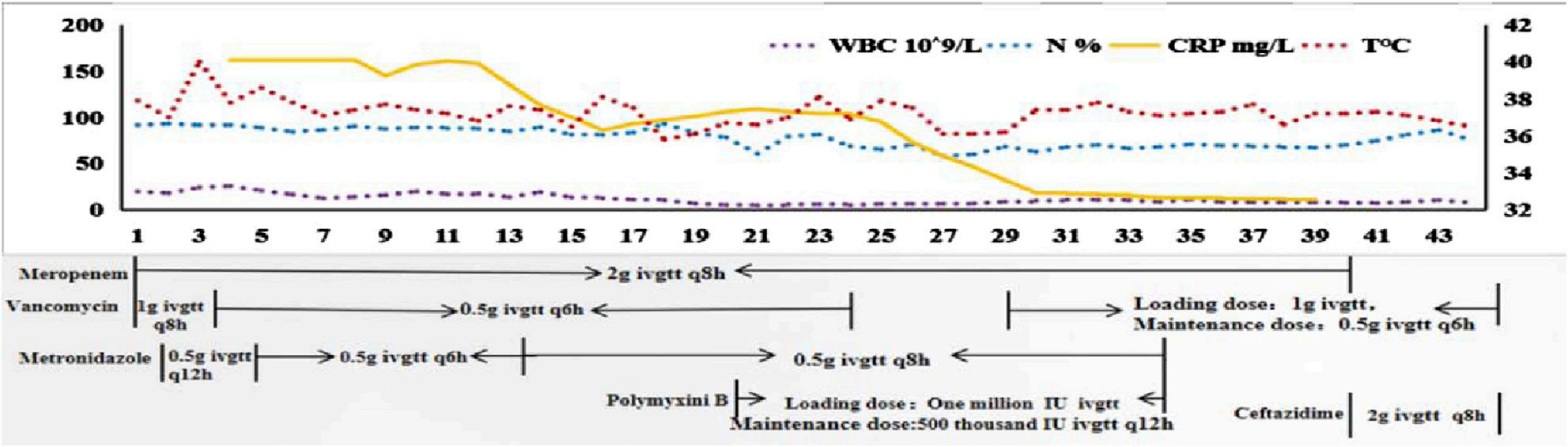
The changes in infection-related indicators and temperature along with the drug use. Note X-axis represents the time of patient admission to the Surgical Intensive Care Unit. WBC, white blood cell; N, neutrophil; CRP, C-reactive protein; T, temperature; ivgtt, intravenous guttae.

**FIGURE 2:**
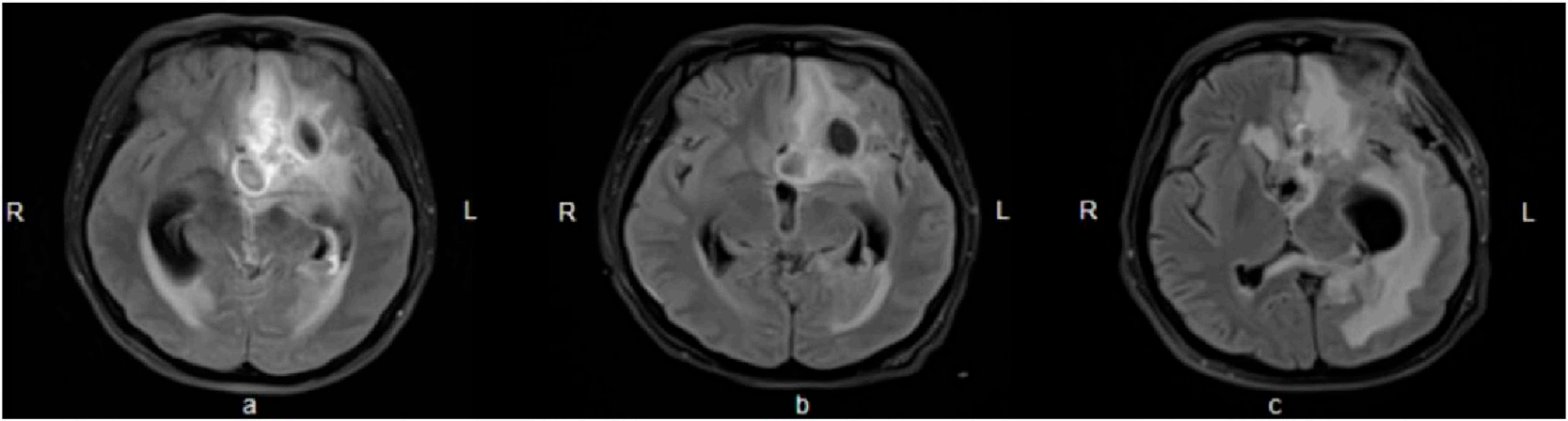
Brain enhanced nuclear magnetic resonance scanning. **(A)** On Day 3, multiple abnormal signals were observed in the left frontotemporal lobe near the base of the skull and in the left lateral paraventricular area, accompanied by peripheral edema, associated with left ventricular ventriculitis and mild leptomeningitis; pus accumulation in the posterior horn of the bilateral ventricles (predominantly on the left side); a slight rightward shift of the midline structures, and left-sided eustachian salpingitis; **(B)** On Day 10, encephalitis with multiple abscesses developed, associated with left ventriculo-ventricular ventriculitis, meningoencephalitis and spongiitis, indicating overall progress compared to the previous period; **(C)** On Day 42, some of the abscesses in the left frontal lobe and left corpus callosum pressure-somatic region had decreased in size compared to earlier assessments. Lateral ventricular ventriculitis and meningitis showed improvement, while ventriculitis in the fourth ventricular region and the rightward shift of midline structures had progressed further.

A 24-year-old woman was admitted to hospital with a fever and headache that had persisted for more than 2 weeks, occurring 32 days post-caesarean section. She had no significant medical history. The patient was diagnosed with puerperal fever, a headache requiring further investigation, and a possible intracranial infection. Two days prior to her admission, she experienced exacerbated vomiting, accompanied by a body temperature reaching 38.5°C. magnetic resonance imaging (MRI) revealed multiple abnormal signal shadows in the left frontal lobe, as well as in the anterior and posterior horns of the left ventricle, raising the consideration of encephalitis as a potential diagnosis. Upon her transfer to our hospital’s Surgical Intensive Care Unit (SICU), the patient exhibited an exacerbation of impaired consciousness and was intubated with an endotracheal tube. The patient’s condition and laboratory examination revealed gingival abscesses, confusion, and an inability to respond to verbal stimuli. Bilateral pupils were equal in size and round (3.5 mm in diameter), with intact light reflexes. The patient’s temperature was recorded at 40°C, with a white blood cell (WBC) count of 24.21 × 10^9^/L, a neutrophil percentage of 93.7%, and a C-reactive protein level exceeding 161 mg/L (Figure 1). Liver function tests were normal, with a creatinine level of 40 μmol/L. Additionally, cranial magnetic resonance imaging of the brain is presented in Figure 2 and CSF results are shown in Table 1. The patient was administered meropenem (2 g q8h ivgtt) and vancomycin (1 g q8h ivgtt) (Figure 1). On Day 3, the patient remained in a coma, unresponsive to verbal stimuli, exhibiting bilaterally unequal pupils, reduced muscle tone in both limbs, and a lack of significant withdrawal response in the limbs following painful stimuli. Therapeutic drug monitoring (TDM) results are presented in Table 2. CSF mNGS identified the following bacterial species: *Porphyromonas gingivalis* (358 reads), *Prevotella heparinolyticus* (344 reads), *Fusobacterium nucleatum* (15 reads), *Parvimonas micra* (15 reads) and *Filifactor alocis* (14 reads). The treatment regimen included administering 0.5 g of metronidazole intravenously every 12 h. On Day 4, a puncture drainage of the ventricular abscess was performed, with the drainage volume detailed in Table 1., The patient’s creatinine level is 48.4 μmol/L, prompting an adjustment of metronidazole to 0.5 g administered intravenously every 6 h. On Day 6, the bacterial culture of CSF revealed the growth of *Prevotella heparinolyticus*. By Day 13, the patient was able to open her eyes, and there was a slight improvement in muscle strength of the extremities: the left lower extremity exhibited grade 3 strength, the right lower extremity exhibited grade 1 strength, and both upper extremities exhibited grade 4 strength. The dosage of metronidazole was adjusted to 0.5 g ivgtt every 8 h. On Day 15, the patient underwent partial resection of multiple intracranial abscesses, bilateral lateral ventricle drainage, right lateral ventricle Ommaya reservoir implantation, and craniotomy for intracranial decompression. On Day 19, *Acinetobacter lwoffii* and *Stenotrophomonas maltophilia*, were isolated from blood culture, prompting the addition of polymyxin B sulfate to the treatment regimen. On Day 23, the patient demonstrated the ability to cooperate when called. CSF and blood cultures consistently tested negative for Gram-positive bacteria, resulting in the discontinued of vancomycin. On Day 27, the patient’s creatinine level was 28 μmol/L. On Day 29, the patient’s creatinine level is 38.9 μmol/L. On Day 31, a chest X-ray revealed a patchy shadow in the right lower lung field, leading to the reinstitution of vancomycin in the treatment regimen. By Day 35, inflammatory indicators showed a decrease compared to previous measurements, which facilitated the discontinuation of polymyxin B and metronidazole. On Day 39, the patient’s creatinine level was 35 μmol/L. On Day 41, the patient was alert with open eyes when called. The left pupil measured 4 mm, and the right pupil also measured 4 mm. Muscle strength in the left lower limb was assessed at grade 1, while the right lower limb was similarly graded at grade 1; both upper limbs exhibited muscle strength at grade 2. Meropenem was discontinued, and treatment continued with ceftazidime and vancomycin. By Day 45, the oxygenation index is unstable and does not meet the criteria for off-boarding, necessitating continued use of the ventilator. Concurrently, ventricular drainage was being administered to manage intracranial pressure. Inflammatory markers were within normal limits, leading to the cessation of all antibiotics, after which the patient was transferred to another hospital for further rehabilitation therapy. After 3 months of follow-up, the patient demonstrated significant recovery, regaining the ability to express language and restoring muscle strength to near normal levels.

**TABLE 1:** Cerebrospinal fluid test results and condition of drainage.

Inspection items	D1	D3	D6	D13	D17	D19	D22	D23	D31	D35	D41
Glucose (mol/L)	<1.1	—	1.41	3.77	4.21	4.69	3.4	—	—	3.19	3.1
Leukocytes (10^6^/L)	9,962	—	1,362	282	185	19	16	—	—	21	16
Chloride (mmol/L)	105	—	116	121	122	124	119	—	—	125	129
Lactate dehydrog- enase (U/L)	—	884	662	626	125	99	—	134	91	33	—
Protein (mg//L)	—	8,902	2,237.2	2,494.6	1,559.9	1,686.6	—	1779	1,227.8	709	—
The color of CSF	light yellow	—	light yellow	light yellow	light yellow	light yellow	light yellow	light yellow	light yellow	light yellow	light yellow
Turbidity	turbid	—	turbid	micro hybrid	micro hybrid	Limpi-dness	Limpi-dness	Limpi-dness	Limpi-dness	Limpi-dness	Limpi-dness
Drainage ventricle 1 (mL)	—	10	20	40	86	100	0	50	35	—	—
Drainage ventricle 2 (mL)	—	50	100	125	96	100	90	70	30	—	—
External ventricular drainage (mL)	—	—	—	—	—	—	—	—	100	145	110

Note D represents the time of patient admission to the Surgical Intensive Care Unit.

CSF, cerebrospinal fluid.

**TABLE 2:** Therapeutic drug monitoring result of vancomycin, meropenem and polymyxin B.

Drug	Day	Therapeutic regimen	C_max_ (μg/mL)	C_min_ (μg/mL)	AUC/MIC	%T > MIC	AUC_ss, 24h_ (mg·h/L)
Vancomycin	D3	1 g ivgtt q8 h Slow drip 2 h	48.87	22	798.8		
	D8	0.5 g ivgtt q6 h Slow drip 2 h	19.82	13.26	384.5		
	D15	0.5 g ivgtt q6 h Slow drip 2 h	19.79	7.57	302.5		
	D36	0.5 g ivgtt q6 h Slow drip 2 h	25.9	19.62	486.9		
	D42	0.5 g ivgtt q6 h Slow drip 2 h	23.21	12.78	411.6		
Meropenem	D3	2 g ivgtt q8 h Slow drip 2 h	28.44	3.45		88.7	
	D7	2 g ivgtt q8 h Slow drip 2 h	49.86	1.34		73.8	
	D14	2 g ivgtt q8 h Slow drip 2 h	57.12	0.19		63.77	
	D29	2 g ivgtt q8 h Slow drip 2 h	27.15	1.69		84.3	
	D31	2 g ivgtt q8 h Slow drip 2 h	46.49	1.84		78.1	
Polymyxin B	D5	50 mg ivgtt q12 h	3.87	1.25			59.1

Note Day represents the time of patient admission to the Surgical Intensive Care Unit.

C_max_, peak serum concentrations; C_min_, trough serum concentrations; AUC/MIC, area under the curve/minimum inhibitory concentration; %T > MIC, the percentage time above the minimal inhibitory concentration; ivgtt, intravenous guttae; AUC_ss, 24h_, area under the concentration-time curve across 24 h at steady state.

## Discussion

The patient was diagnosed with an anaerobic brain abscess and achieved a favorable clinical outcome through surgical resection and aggressive anti-infective treatment, which included a combination of antimicrobial agents, specifically meropenem and metronidazole.

Anaerobic infections often present as simultaneous infections involving various anaerobic bacteria or in combination with aerobic bacteria. The increasing antibiotic resistance of these bacteria to antimicrobial drugs complicates the treatment of associated infections. Standard treatment for anaerobic abscess bacteria involves antimicrobial therapy, which serves as a crucial adjunct to drainage and surgical interventions. Administering antimicrobials prior to drainage can reduce culture sensitivity. However, this treatment should not be postponed while awaiting drainage. Since the mixed infection of anaerobic and aerobic bacteria was quite common, the selected antimicrobial agent should effectively target both. Our patient was treated with a combination of vancomycin and meropenem for the management of infection empirically. A dose of vancomycin, 1 g every 8 h, was administered to rapidly achieve the desired blood concentration.

Meropenem is classified as a time-dependent antibiotic, with its pharmacokinetic/pharmacodynamic (PK/PD) parameters characterized by the percentage of the dosing interval during which the drug concentration exceeds the minimum inhibitory concentration (%T > MIC) (Mattoes et al., 2004). Zhang et al. (2017) investigated the administration of meropenem at doses of 1 g every 6 h and 2 g every 8 h in patients with postoperative meningitis following neurosurgery, reporting treatment success rates of 88.1% and 94.7%, respectively. In a study conducted by Hiroshige Mikamo et al. (2008) on PK-PD of meropenem, no correlation was found between T > MIC values and clinical outcomes in cases of monomicrobial infections caused by anaerobes presenting as abscesses. Conversely, in instances of multimicrobial infections involving both anaerobes and aerobes, clinical efficacy exceeding 90% was achieved with a T > MIC value of 20%. This suggests that the protocols derived from PK-PD theory for treating anaerobic infections are indeed applicable. In this case, the patient presented with mixed anaerobic infections, but the potential for combined aerobic infections could not be ruled out. Meropenem 2 g was administered every 8 h over a 2-h infusion period. Following TDM of meropenem, calculations were performed based on the susceptibility breakpoint of meropenem against Gram-negative bacteria, with a MIC of 4 mg/L. The percentage of time above the MIC (%T > MIC) was found to exceed the target value. This study did not provide the antimicrobial susceptibility results of meropenem for anaerobes, but epidemiological data indicated that certain anaerobes exhibited high MIC values concerning the susceptibility breakpoints of meropenem. Therefore, this study underscores the necessity of enhancing treatment protocols for anaerobic infections.

Novak et al. (2015) demonstrated that metronidazole remains useful antimicrobials for empiric treatment of anaerobic infections, while carbapenems should be reserved forsituations were multidrug resistant, aerobic or facultative Gram-negative bacteria are expected. Liu et al. (2008) reported *that Fusobacterium* spp. demonstrated susceptibility to metronidazole*,* with a resistance rate of 8% to meropenem. A study conducted in the Netherlands showed that *Prevotella* spp. was susceptible to meropenem, reporting a resistance rate to metronidazole of 3% (Veloo et al., 2015). Aldridge et al. (2001) found that *Porphyromonas gingivalis* was susceptible to both metronidazole and meropenem. The findings of A C M Veloo et al. (2011) indicated the identification of one strain of *Parvimonas micra* that was resistant to metronidazole (MIC >256 mg/L), with the MIC_50_ and MIC_90_ values for meropenem reported as 0.047 mg/L and 0.19 mg/L, respectively. Reports on antimicrobial drug resistance in *Filifactor alocis* are scarce. Romero-Martínez et al. (2023) utilized E-test strips to assess the resistance of eight antibiotics, demonstrating that *Filifactor alocis* was generally susceptible to common antibiotics, with metronidazole exhibiting a particularly high degree of susceptibility. However, this article did not address revealing the susceptibility of *Filifactor alocis* to meropenem. Metronidazole exhibits excellent penetration into brain abscesses, with concentrations within these abscesses approximating those found in serum. In this patient, for the possibility of mixed intracranial infections and the potential for partial resistance of meropenem or metronidazole to anaerobic bacteria, a combination regimen of meropenem and metronidazole was employed, effectively managing the patient’s condition.

Metronidazole is a concentration-dependent antibiotic commonly utilized in the treatment of brain abscesses, administered at a dosage of 7.5 mg/kg (typically 500 mg) every 6–8 h, with a maximum allowable dose of 4 g per day. Sprandel et al. (2004) investigated the mean plasma area under the concentration-time curve (AUC_0-24_) for the regimens of 500 mg every 24 h and 500 mg every 8 h regimens, indicating that there was no significant difference between these two regimens; however, both exhibited AUC_0-24_ values that were significantly higher than those observed for the 1,000 mg every 24 h regimen. In this case, the patient began treatment with metronidazole at a dosage of 0.5 g every 12 h, which was subsequently increased to 0.5 g every 6 h due to inadequate dosing. Later, the patient exhibited body tremors, which raised concerns about a potential central nervous system adverse reaction, prompting an adjustment of the dosage to 0.5 g every 8 h. Notably, the therapeutic dosage remained within the recommended range. R. Sonneville et al. (Thy et al., 2022) suggest an empirical treatment cycle of 6 weeks for brain abscesses using metronidazole in conjunction with other medications. However, it is essential to consider the neurotoxic effects associated with this drug, and metronidazole may be discontinued once anaerobic infections are excluded. Common clinical manifestations of metronidazole include limb incoordination, unsteady gait, impaired consciousness, and seizures; however, most patients experience recovery following the discontinuation of the medication. Although metronidazole-induced encephalopathy is a rare complication associated with prolonged use, it is often reversible. The likelihood of developing metronidazole-induced encephalopathy appears to correlate directly with the duration of treatment. Knorr et al. (2012) reported that neurological symptoms emerged after 2–4 weeks of continuous metronidazole use, with cumulative doses ranging from 21 g to 182 g. Upon discontinuation of metronidazole, the symptoms associated with brain disease were completely reversed, and MRI findings returned to baseline. Similarly, AlDhaleei et al. (2018) documented neurotoxic reactions in patients receiving metronidazole (500 mg every 8 h) for over 10 weeks, noting a gradual improvement of symptoms within 4 days following the cessation of the drug. In our patient, metronidazole was administered for a total of 33 days, resulting in a cumulative dose of 53.5 g. Although the duration and total dose of metronidazole fall within the range known to induce adverse reactions in the central nervous system, the patient’s tremor symptoms resolved spontaneously during the course of treatment. This resolution may be attributed to the presence of high intracranial pressure and the underlying conditions associated with the brain abscess. Therefore, we conclude that the symptoms are unlikely to be related to the use of metronidazole.

The safety concerns associated with the high-dose and long-term use of meropenem necessitate careful consideration. Anaka et al. (Tanaka et al., 2013) conducted a study involving 745 patients treated with meropenem, identifying four cases of neurotoxicity; however, cranial MRI or computed tomography (CT) scans showed no abnormalities. Li et al. (2018) indicated in their pharmacokinetic study of meropenem that increasing the intravenous dose may lead to adverse drug reactions, suggesting that prolonging the infusion time could help mitigate the need for higher intravenous doses; however, the specific intravenous dose threshold that may induce epilepsy remains unestablished. Helweg-Larsen et al. (2012) reported a treatment duration of 6–8 weeks for bacterial brain abscesses. In our patient, meropenem was administered via slow drip over 2-h intervals for a total of 6 weeks, during which no seizure symptoms were observed. Overall, meropenem appears to be a relatively safe option for the treatment of central nervous system disorders.

## Conclusion

This study presents a case of a brain abscess in which five species of oral anaerobic bacteria were identified through cerebrospinal fluid mNGS during the puerperium. A combined regimen of meropenem and metronidazole was implemented, and an individualized anti-infection treatment plan was developed based on TDM, providing valuable insights for the management of brain abscesses caused by oral anaerobic bacteria. However, the study’s focus on individual cases limits its generalizability, as the concentration of metronidazole could not be measured, and there was no susceptibility result of meropenem and metronidazole for the various anaerobic bacteria with respect to meropenem and metronidazole. Furthermore, the available clinical research data are quite limited. Therefore, larger clinical studies and reports are necessary to provide substantial data support.

## Data Availability

The original contributions presented in the study are included in the article/supplementary material, further inquiries can be directed to the corresponding authors.

## Abbreviations

CSF, cerebrospinal fluid; NMR, nuclear magnetic resonance; CT, computed tomography; TDM, therapeutic drug monitoring; mNGS, metagenomic next-generation sequencing; SICU, Surgical Intensive Care Unit; CRP, C-reactive protein; WBC, white blood cell; N, neutrophil; T, temperature; ivgtt, intravenous guttae; C_max_, peak serum concentrations; C_min_, trough serum concentrations; AUC/MIC, area under the curve/minimum inhibitory concentration; %T > MIC, the percentage time above the minimal inhibitory concentration; AUC_ss_24h, area under the concentration-time curve across 24 h at steady state.

## References

[B1] AlDhaleei W. AlMarzooqi A. Gaber N. (2018). Reversible metronidazole-induced neurotoxicity after 10 weeks of therapy. BMJ case Rep. 2018, bcr2017223463. 10.1136/bcr-2017-223463 PMC5911128 29678819

[B2] Aldridge K. E. Ashcraft D. Cambre K. Pierson C. L. Jenkins S. G. Rosenblatt J. E. (2001). Multicenter survey of the changing *in vitro* antimicrobial susceptibilities of clinical isolates of Bacteroides fragilis group, Prevotella, Fusobacterium, Porphyromonas, and Peptostreptococcus species. Antimicrob. agents Chemother. 45 (4), 1238–1243. 10.1128/aac.45.4.1238-1243.2001 11257040 PMC90449

[B3] Bodilsen J. Mariager T. Duerlund L. S. Storgaard M. Larsen L. Brandt C. T. (2024). Brain abscess caused by oral cavity bacteria: a nationwide, population-based cohort study. Clin. Infect. Dis. official Publ. Infect. Dis. Soc. Am. 78 (3), 544–553. 10.1093/cid/ciad678 37946527

[B4] Helweg-Larsen J. Astradsson A. Richhall H. Erdal J. Laursen A. Brennum J. (2012). Pyogenic brain abscess, a 15 year survey. BMC Infect. Dis. 12, 332. 10.1186/1471-2334-12-332 23193986 PMC3536615

[B5] Knorr J. P. Javed I. Sahni N. Cankurtaran C. Z. Ortiz J. A. (2012). Metronidazole-induced encephalopathy in a patient with end-stage liver disease. Case Rep. hepatology 2012, 209258. 10.1155/2012/209258 PMC4208391 25374704

[B6] Li X. Sun S. Wang Q. Zhao Z. (2018). Population pharmacokinetics of combined intravenous and local intrathecal administration of meropenem in aneurysm patients with suspected intracranial infections after craniotomy. Eur. J. drug metabolism Pharmacokinet. 43 (1), 45–53. 10.1007/s13318-017-0422-1 28616823

[B7] Liu C. Y. Huang Y. T. Liao C. H. Yen L. C. Lin H. Y. Hsueh P. R. (2008). Increasing trends in antimicrobial resistance among clinically important anaerobes and Bacteroides fragilis isolates causing nosocomial infections: emerging resistance to carbapenems. Antimicrob. agents Chemother. 52 (9), 3161–3168. 10.1128/aac.00355-08 18625771 PMC2533455

[B8] Mattoes H. M. Kuti J. L. Drusano G. L. Nicolau D. P. (2004). Optimizing antimicrobial pharmacodynamics: dosage strategies for meropenem. Clin. Ther. 26 (8), 1187–1198. 10.1016/s0149-2918(04)80001-8 15476901

[B9] Mikamo H. Yamagishi Y. Tanaka K. Watanabe K. (2008). Clinical investigation on target value of T>MIC in carbapenems. Jpn. J. antibiotics 61 (2), 73–81. 18669417

[B10] Novak A. Rubic Z. Dogas V. Goic-Barisic I. Radic M. Tonkic M. (2015). Antimicrobial susceptibility of clinically isolated anaerobic bacteria in a University Hospital Centre Split, Croatia in 2013. Anaerobe 31, 31–36. 10.1016/j.anaerobe.2014.10.010 25479237

[B11] Romero-Martínez R. Maher A. Àlvarez G. Figueiredo R. León R. Arredondo A. (2023). Whole genome sequencing and phenotypic Analysis of antibiotic resistance in filifactor alocis isolates. Antibiot. Basel, Switz. 12 (6), 1059. 10.3390/antibiotics12061059 PMC10295267 37370380

[B12] Sonneville R. Ruimy R. Benzonana N. Riffaud L. Carsin A. Tadié J. M. (2017). An update on bacterial brain abscess in immunocompetent patients. Clin. Microbiol. Infect. official Publ. Eur. Soc. Clin. Microbiol. Infect. Dis. 23 (9), 614–620. 10.1016/j.cmi.2017.05.004 28501669

[B13] Sprandel K. A. Schriever C. A. Pendland S. L. Quinn J. P. Gotfried M. H. Hackett S. (2004). Pharmacokinetics and pharmacodynamics of intravenous levofloxacin at 750 milligrams and various doses of metronidazole in healthy adult subjects. Antimicrob. agents Chemother. 48 (12), 4597–4605. 10.1128/aac.48.12.4597-4605.2004 15561831 PMC529226

[B14] Takahashi M. Nakanishi Y. Hamada Y. Hoshimoto Y. Aoki J. Karakida K. (2020). A case of brain abscess caused by actinomyces cardiffensis and Parvimonas micra. Tokai J. Exp. Clin. Med. 45 (4), 189–194. 33300589

[B15] Tanaka A. Takechi K. Watanabe S. Tanaka M. Suemaru K. Araki H. (2013). Comparison of the prevalence of convulsions associated with the use of cefepime and meropenem. Int. J. Clin. Pharm. 35 (5), 683–687. 10.1007/s11096-013-9799-3 23733559

[B16] Thy M. Gaudemer A. d'Humières C. Sonneville R. (2022). Brain abscess in immunocompetent patients: recent findings. Curr. Opin. Infect. Dis. 35 (3), 238–245. 10.1097/qco.0000000000000833 35665718

[B17] Veloo A. C. Boiten K. E. Wekema-Mulder G. J. Rurenga P. Singadji Z. M. Scoop G. G. (2015). Antibiotic susceptibility profiles of Prevotella species in The Netherlands. Int. J. Antimicrob. agents 45 (5), 554–556. 10.1016/j.ijantimicag.2015.01.003 25726442

[B18] Veloo A. C. Welling G. W. Degener J. E. (2011). Antimicrobial susceptibility of clinically relevant Gram-positive anaerobic cocci collected over a three-year period in The Netherlands. Antimicrob. agents Chemother. 55 (3), 1199–1203. 10.1128/aac.01771-09 21189338 PMC3067104

[B19] Zhang Y. Zhang J. Chen Y. Yu J. Cao G. Wu X. (2017). Evaluation of meropenem penetration into cerebrospinal fluid in patients with meningitis after neurosurgery. World Neurosurg. 98, 525–531. 10.1016/j.wneu.2016.11.040 27867128

[B20] Zhong X. Wang M. Meng Q. Jiang X. Guo Z. Zhang Y. (2023). Meningitis caused by oral anaerobes detected using mNGS tool: a case report and review of literature. BMC Neurol. 23 (1), 344. 10.1186/s12883-023-03307-2 37775739 PMC10542268

